# Tracking diphyodont development in miniature pigs *in vitro* and *in vivo*

**DOI:** 10.1242/bio.037036

**Published:** 2019-01-25

**Authors:** Fu Wang, Guoqing Li, Zhifang Wu, Zhipeng Fan, Min Yang, Tingting Wu, Jinsong Wang, Chunmei Zhang, Songlin Wang

**Affiliations:** 1 Molecular Laboratory for Gene Therapy & Tooth Regeneration, Beijing Key Laboratory of Tooth Regeneration and Function Reconstruction, School of Stomatology, Capital Medical University, Beijing 100050, China; 2 Department of Basic Oral Sciences, School of Stomatology, Dalian Medical University, Dalian 116044, China; 3 Laboratory of Molecular Signaling and Stem Cells Therapy, Beijing Key Laboratory of Tooth Regeneration and Function Reconstruction, School of Stomatology, Capital Medical University, Beijing 100050, China; 4 Department of Biochemistry and Molecular Biology, School of Basic Medical Sciences, Capital Medical University, Beijing 100069, China

**Keywords:** Diphyodont development, Successional tooth formation, Tooth replacement, Large animal, Wuzhishan pig

## Abstract

Abnormalities of tooth number in humans, such as agenesis and supernumerary tooth formation, are closely related to diphyodont development. There is an increasing demand to understand the molecular and cellular mechanisms behind diphyodont development through the use of large animal models, since they are the most similar to the mechanism of human tooth development. However, attempting to study diphyodont development in large animals remains challenging due to large tooth size, prolonged growth stage and embryo manipulation. Here, we characterized the expression of possible genes for diphyodont development and odontogenesis of an organoid bud from single cells of tooth germs *in vitro* using *Wzhishan pig* strain (*WZSP*). Following this, we used a method of ectopic transplantation of tooth germs at cap stage to dynamically track diphyodont development of tooth germs in mouse subrenal capsules to overcome the restrictions in pig embryos. The results showed that pig tooth germ at cap stage could restore diphyodont development and maintain efficient long-term survival and growth in mouse subrenal capsules, which is suitable for future manipulation of large mammalian tooth development. Our pilot study provided an alternative for studying diphyodont development in large mammals, which will further promote the use of pig as a diphyodont model similar to humans for craniofacial development study.

## Abstract (precis)

**Summary:** Little is known about diphyodont development in large animals. Our pilot trial characterized this gene expression and developed an alternative method to track diphyodont development in pigs.

## INTRODUCTION

Mammals have lost the ability for continuous tooth renewal seen in most other vertebrates, and typically have only one or two generations of teeth. Unlike monophyodont mice and polyphyodont fish and reptiles, humans and most mammals belong to diphyodont type of dentition (two sets of teeth) with a deciduous (primary) set of 20 teeth and a permanent set of 28–32 teeth. The permanent incisors, canines and premolars are successional teeth which arise from the extension of the dental lamina on the lingual aspect of the deciduous teeth and replace their primary counterpart.

The abnormalities of tooth number such as tooth agenesis (hypodontia, oligodontia and anodontia) and formation of extra teeth in humans are closely related to diphyodont development (Juuri and Balic, 2017). Though molecular genetics has provided great progress in tooth agenesis research, and many genes have been identified that are involved in tooth agenesis, very little is known of molecular and cellular mechanisms governing this process (Juuri and Balic, 2017; Yin and Bian, 2015). Currently, understanding diphyodont development is hindered partly by a lack of models that faithfully simulate human tooth development and longer developmental periods required for tooth development and replacement in humans. The mouse, the main mammalian model for development without tooth replacement and all tooth classes, is not an ideal model for understanding tooth replacement (Tucker and Fraser, 2014). Some polyphyodont species, such as fish and reptiles, have been adopted to study the tooth renewal (Fraser et al., 2013; Handrigan et al., 2010; Juuri and Balic, 2017; Richman and Handrigan, 2011; Wu et al., 2013). However, polyphyodont species replace their teeth throughout life and have more simple shapes which are different from diphyodont species. More recently, diphyodont species such as ferrets and pigs are used to offer new insights into tooth replacement mechanisms (Järvinen et al., 2009; Wang et al., 2014a,b). The ferret, as a model for studying diphyodont replacement, has some inconveniences because they have a seasonal estrus, and therefore embryos cannot be collected continuously.

Pigs (*Sus Scrofa*) serve as a promising large animal model for studying human diseases and contribute to overcoming the shortage of human donor organs (Kemter and Wolf, 2015; Wu et al., 2017). The miniature pig has proven to be a valuable animal model for diphyodont development due to its many similarities to humans, including the morphology, number and size of teeth, particularly its heterodont dentition (incisors, canines, premolars and molars) and diphyodont dentition, which are not available in rodents (Wang et al., 2007). The morphology and chronology of diphyodont dentition in Wuzhishan pig strain (*WZSP*) have been well characterized by our previous studies (Wang et al., 2014a). We also obtained the profiling and functional network of differential gene expression during early diphyodont development from *WZSP*. Moreover, recent breakthrough in porcine genome engineering aiming to overcome immunological challenges and potential risk of PERV transmission make safe clinical xenotransplantation possible (Ekser et al., 2012; Niu et al., 2017; Wu et al., 2017). However, due to ethical concerns, scientific reasons, expense and other considerations, dynamic tracking of early diphyodont development *in vivo* in pig remains a significant obstacle. Some issues need to be overcome, such as longer process required for growth of swine teeth with larger size, inconvenient embryo obtaining and detection. There is an increasing demand to seek alternative approaches for studying diphyodont development in pigs.

The transplantation of graft under the renal capsule is often used for the study of tissue development and organogenesis, however, it remains unclear whether the renal capsule microenvironment can sustain the long-term growth of tooth germs from pig. Moreover, recent modeling organogenesis using organoid culture technology by cell assembly in 3D environment can allow cells to recapitulate cell interactions and generate organ-like tissues occurring during organogenesis *in vivo* and promise alternatives for understanding diphyodont development and replacement in pig (Bredenoord et al., 2017; Camp et al., 2017; Pennisi, 2017; Schweiger and Jensen, 2016).

Here, we successfully achieved long-term survival and growth of *WZSP* tooth germs through ectopic transplantation in mouse subrenal capsules, which contribute to dynamically tracking diphyodont development of large animal. Our pilot study provides alternative methods to study diphyodont development and replacement of large mammal models, which will further promote the use of pig as a diphyodont model similar to humans.

## RESULTS

### The expression of genes during diphyodont development at early stage

Our previous study suggested that successional dental lamina appears when primary tooth germ reaches early bell stage in *WZSP* and screened some differentially expressed genes during early diphyodont development (Wang et al., 2014a,b). We chose the forth deciduous molar germs (p4) of *WZSP* as the research object, which was at early bell stage, bell stage and secretory stage respectively at E50, E60 and/or E70. The early successional dental laminae was initiated after E50 (Fig. S1).

**Fig. 1.**
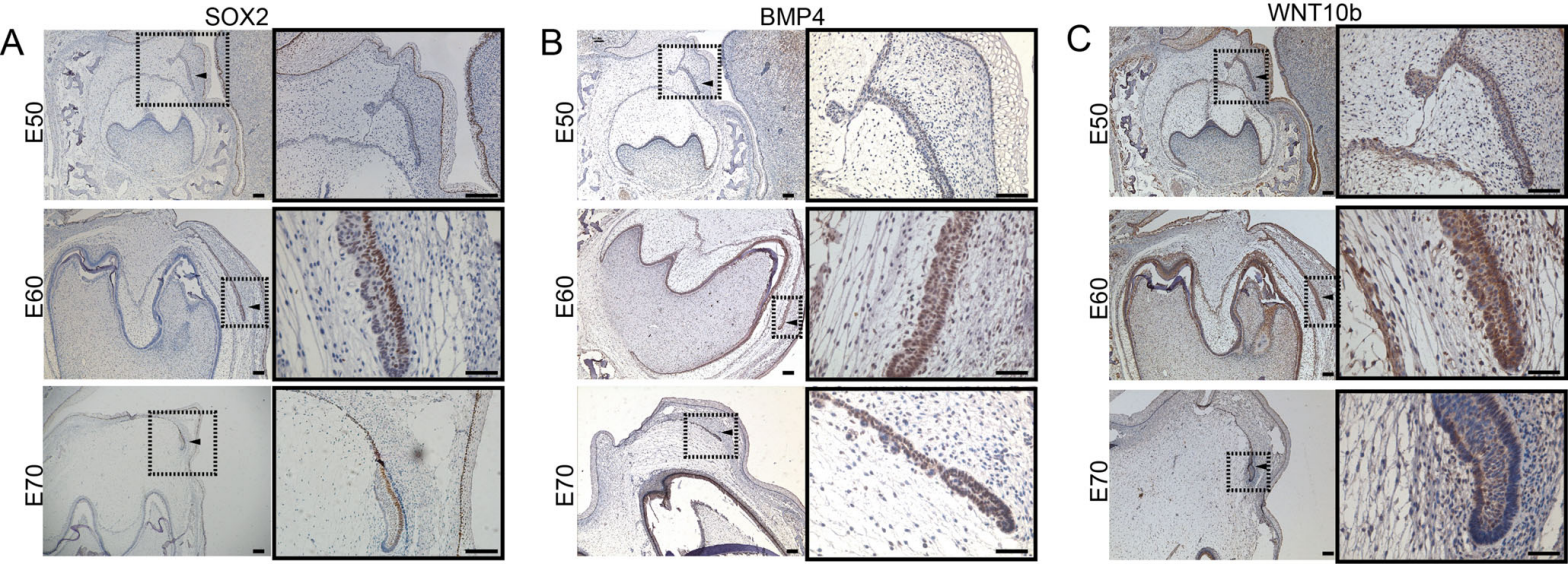
**The expression of candidate genes in the developing mandibular p4 and successional dental lamina in frontal sections of *WZSP* embryo.** (A) SOX2, (B) BMP4 and (C) WNT10b immunohistochemical stain. (A–C) Right panels show the enlarged successional dental lamina, shown with an arrowhead inside the boxed area in the left panels. Scale bars: 500 μm (left panels), 50 μm (right panels).

We first investigated the differentially expressed SOX2, BMP4 and WNT10b during early diphyodont development based on the findings of our previous study by immunohistochemical analysis (Wang et al., 2014b). Sox2 represents a marker of epithelial competence during tooth generation in mammals (Juuri et al., 2012). We found that Sox2 was specifically expressed in lingual epithelium of the successional dental lamina. In contrast, no specific expression was seen in the region of p4 germ (Fig. 1A). There was also strong expression of SOX2 in the oral epithelium (Fig. 1A). BMP4 expression predominated in the inner enamel epithelium of p4 and successional dental lamina at E50, then was strongly expressed in the inner enamel epithelium, dental papilla and successional dental lamina at E60 and E70 (Fig. 1B). The strong expression domains of WNT10b were seen in the inner and outer enamel epithelium, enamel knot of p4, successional dental lamina and oral epithelium from E50 to E70. WNT10b also were weakly expressed in dental papilla at E60 and E70 (Fig. 1C).

**Fig. 2.**
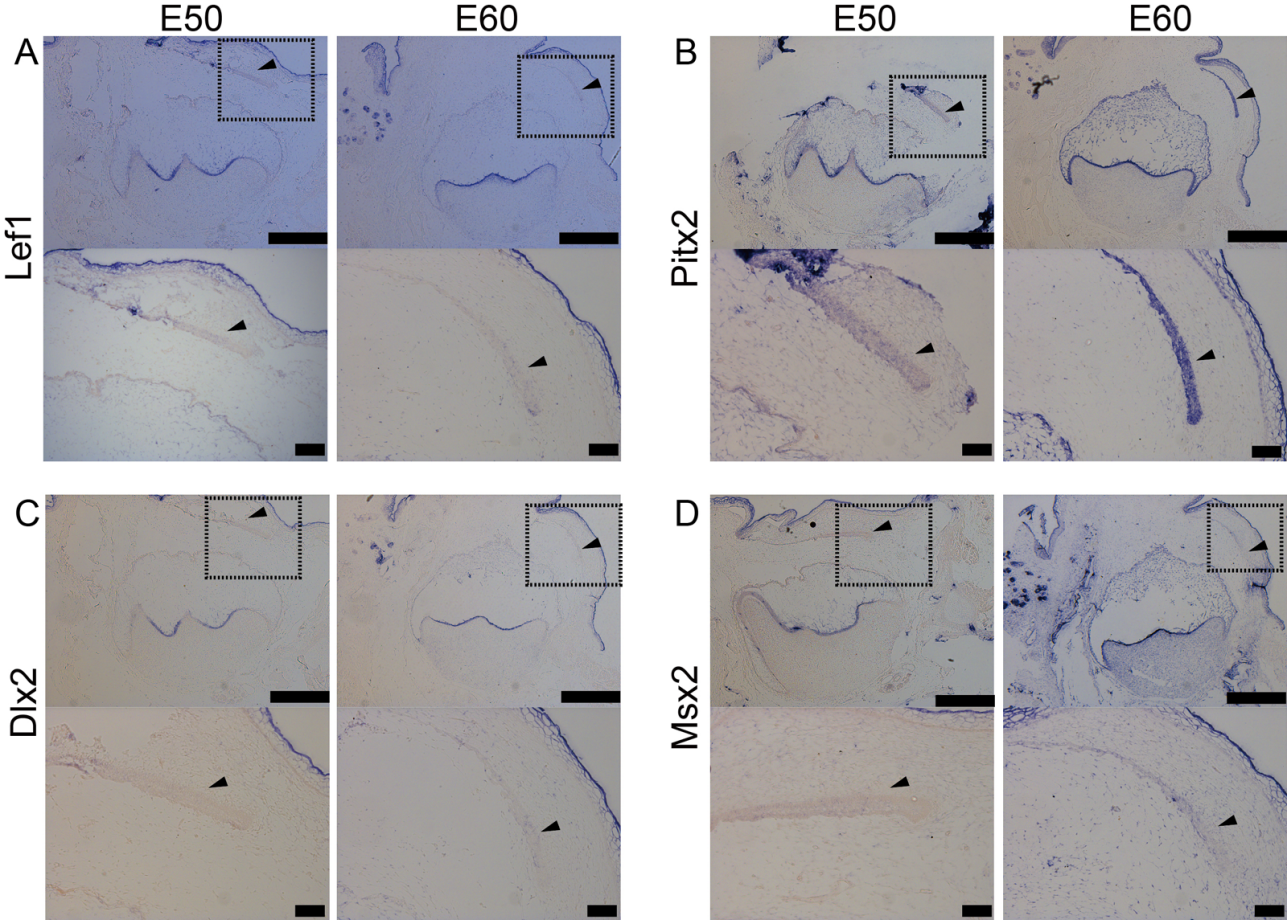
***In situ* hybridization analysis of transcript factor expression in developing mandibular p4 and successional dental lamina at E50 and E60.** (A) The expression of *Lef1*. (B) The expression of *Pitx2*. (C) The expression of *Dlx2.* (D) The expression of *msx2*. (A–D) Lower panels show the enlarged successional dental lamina, shown with an arrowhead inside the boxed area in the upper panels. Scale bars: 500 μm (upper panels), 50 μm (lower panels).

Given that the specific transcription factors associated with the dental identity have crucial roles in teeth development and connection to human disease (Balic, 2019; Balic and Thesleff, 2015; Fournier et al., 2018), we further investigated the gene expression of *Left1*, *Pitx2*, *Dlx2* and *Msx2* at E50 and E60 by *in situ* hybridization after the successional dental lamina was initiated. All of them were strongly expressed in the inner enamel epithelium of p4 and oral epithelium both at E50 and E60, and weakly expressed in successional dental lamina (Fig. 2A–D), except for strong expression of *Pitx2* in successional dental lamina at E60 (Fig. 2B). The expression of *Left1* and *Pitx2* in dental papilla of p4 were stronger at E60 than it at E50 (Fig. 2A,B), while, the expression of *Dlx2* and *Msx2* in dental papilla of p4 was very low at E50 and upregulated at E60 (Fig. 2C,D).

### Self-organized cell spheroids of single tooth germ cells in suspension culture

**Fig. 3.**
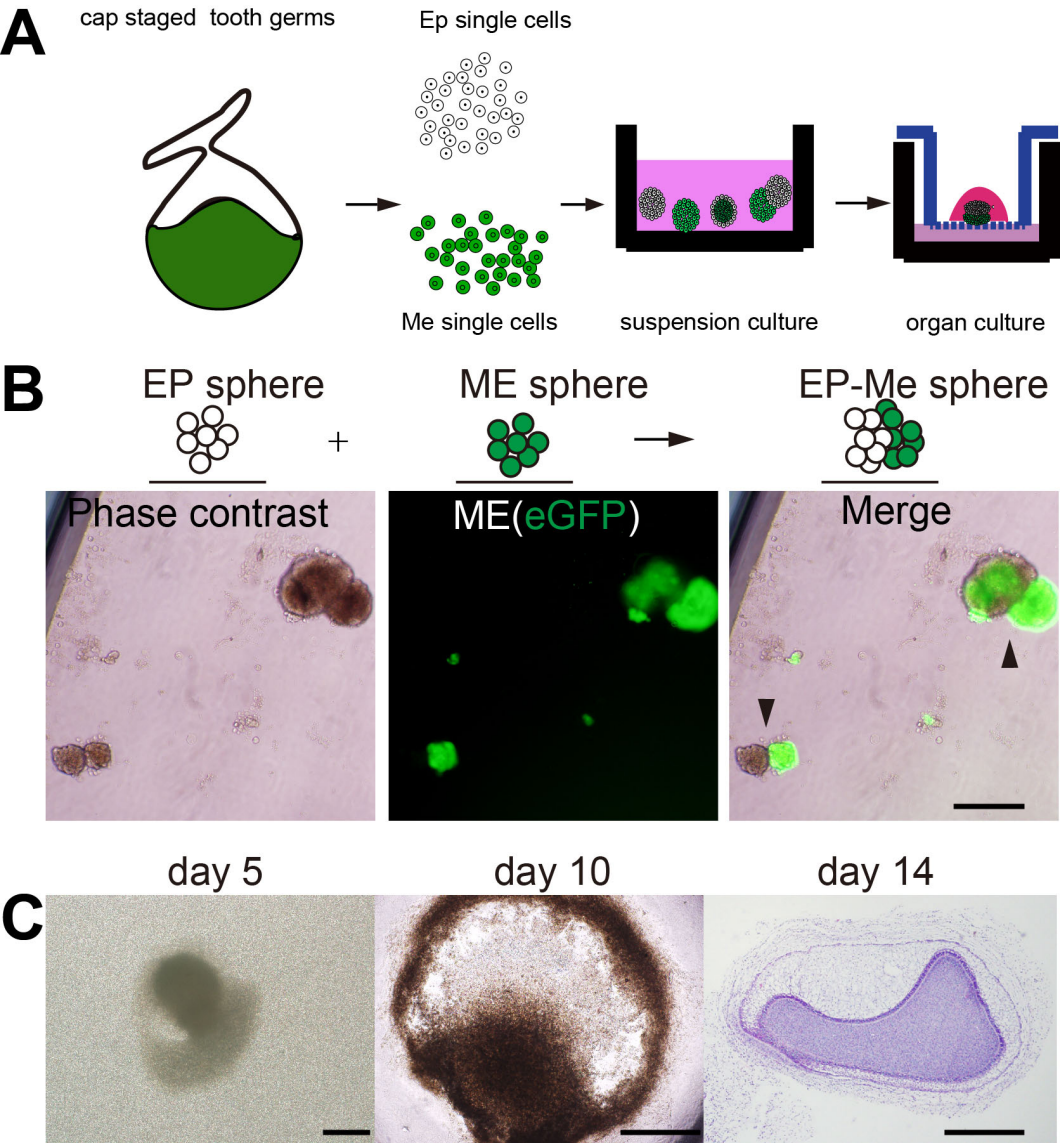
**Self-organized cell spheroids from single tooth germ cells in suspension culture.** (A) Schematic diagram of experimental design. (B) Single cells from tooth germs in ultra-low-attachment plates self-assembled to form epithelial cell spheres (Ep sphere, left), mesenchymal cell spheres (Me-sphere, marked with eGFP, middle), mixed epithelial and mesenchymal spheres self-organized to form interaction of cell spheroids (Ep-Me sphere, arrowheads, right). Scale bar: 500 μm. (C) The interactive cell spheroids in organ culture and histological sections showing morphogenesis in organ culture (H&E). Scale bars: 200 μm.

**Fig. 4.**
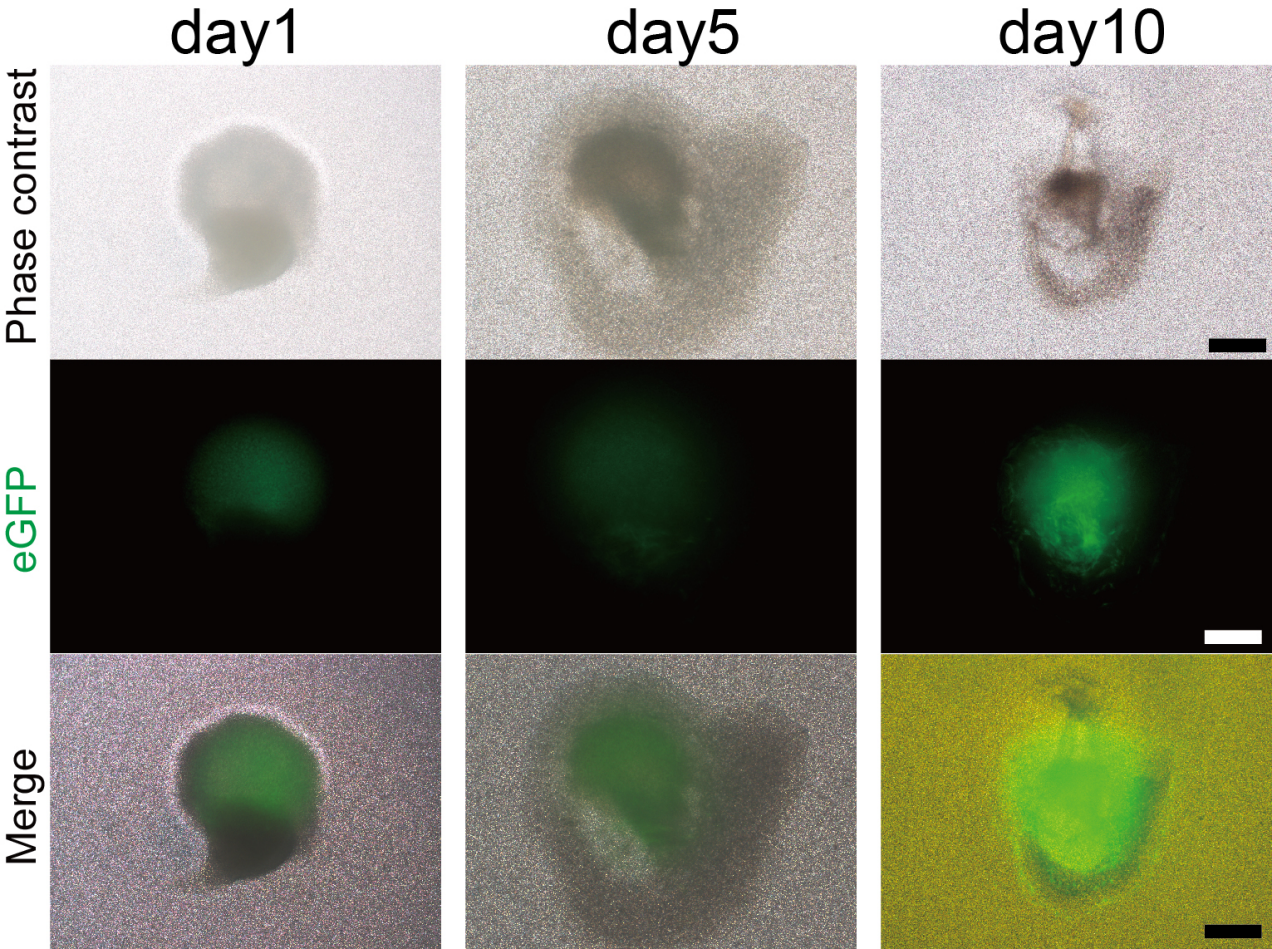
**Tracking the morphogenesis of a reassociated organoid from single tooth germ cells at cap stage with epithelial cell and mesenchymal cell compartment in organ culture.** The morphogenesis of organoid in organ culture (mesenchymal cell marked with eGFP, green). Scale bars: 500 µm.

There is a great need of pregnant *WZSP*s and embryos for constructing re-associations that allow for assessment of tooth regeneration strategies. To reduce the high number of animal experiments, we introduced a high-efficiency protocol to generate cell spheroids to facilitate applications for tissue engineering (Fig. 3A). Thus, we tested single cells’ self-organization in suspension culture in ultra-low-attachment microplates, which can inhibit cellular attachment and contribute to the formation of cell spheroids, to characterize interactions between single cells (Sasai, 2013). After transfer into ultra-low-attachment 12-well plates, single epithelial or mesenchymal cells from tooth germ were able to self-aggregate to form many epithelial cell spheroids (Ep sphere) or mesenchymal cell spheroids (Me sphere) (Fig. 3B). To generate interactions between cell spheroids, we mixed spheroids made of epithelial and mesenchymal cells in ultra-low-attachment plates. The contact between two types of spheroids were observed (Ep-Me sphere) (Fig. 3B). The Ep-Me organoids were transferred into transwell insert for organ culture, which also restored the *de novo* odontogenesis, histological sections showing morphogenesis in organ culture (Fig. 3C). To further track the epithelial-mesenchymal interactions, the mesenchymal cells were marked with eGFP. We could track the morphogenesis of reassociated organoid from single tooth germ cells in organ culture (Fig. 4).

### Growth and survival of intact tooth germ in subrenal capsules of SCID mice

**Fig. 5.**
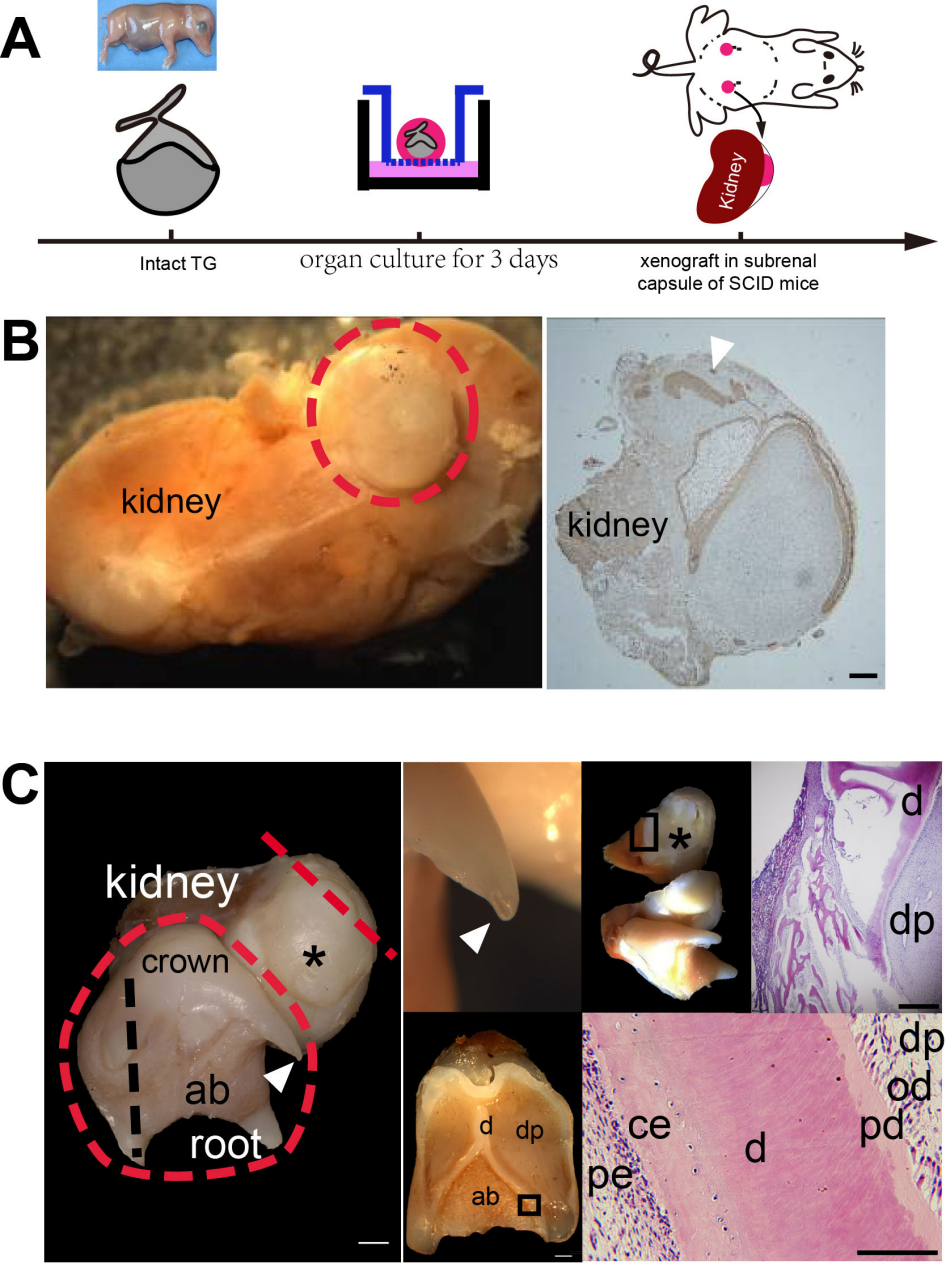
**The tooth germ at cap stage restored diphyodont development in mouse subrenal capsules.** (A) Schematic representation of obtaining *WZSP*s' tooth germ at cap stage and ectopic transplantation in the mouse subrenal capsules of nude mice. (B) A successional dental lamina was initiated when an intact deciduous molar germ at the cap stage was transplanted after 2 weeks (*n*=3). Left panel shows macro view of regenerated tissues (red dotted line). Right panel shows immunohistochemistry analysis (anti-CK14) showed a successional dental lamina (arrowhead). (C) One intact tooth germ at cap stage developed into a primary deciduous molar and replacement tooth (asterisk) at 16 weeks post-transplantation in mouse subrenal capsules. Left panel shows macro view of a regenerated deciduous molar (red dotted line) with typical crown, root, apical foramens (arrowhead) of deciduous molar. Upper right panels show enlarged apical foramens (arrowhead), cross view of replacement tooth (asterisk) at the red dotted line in the left panel and histological analysis (H&E) of the boxed area. Lower right panels show cross view of primary deciduous molar at the black dotted line in the left panel and histological analysis (H&E) of the boxed area. ab, alveolar bone; am, ameloblast; ce, cementum; d, dentin; dp, dental pulp; e, enamel; od, odontoblast; pd, predentin; pe, periodontium; rt, renal tissue. Scale bars: cross view, 1 mm; staining, 100 µm.

The *WZSP*’s deciduous molars are similar to humans; both are larger and require a longer developmental period than those of mice. Our previous results showed that approximately 3 months were required to grow p4 from the cap stage at E40 to eruption with nearly completed roots at postnatal day 20 (P20) (Wang et al., 2014a). It is critical to develop an environment using alternative methods for long term observation for large size tooth growth. Whether the subrenal capsules of mice are sufficient to sustain the growth and survival of remains unknown. We tested feasibility *in vivo* for growth of tooth germ with long developmental period under renal capsules (Fig. 5A). When an intact deciduous molar germ at the cap stage after organ culture for 3 days *in vitro* was transplanted into subrenal capsules, the tooth germ continued to develop. In this study, a total of nine grafts were performed. Three out of nine grafts were observed for only 2 weeks, all of which formed the successional dental lamina viewed by histochemical section (Fig. 5B); four out of the other six grafts grew into a deciduous molars with a replacement tooth at 16 weeks post-transplantation with normal morphology, including crown, roots and supporting tissues (periodontium, cementum and attached bone) (Fig. 5C). Surprisingly, in one of the grafts, the four roots, as well as the apical foramens of a deciduous molar, were clearly visible. Serial sections revealed that the crown attached to the mouse kidney with correct tooth structure, including all dental components (Fig. 5C).

## DISCUSSION

In the present study, we characterized the expression of candidate genes for diphyodont development and odontogenesis of organoid bud from single cells of pig tooth germs *in vitro*, then used a method of ectopic transplantation of tooth germs at cap stage to dynamically track diphyodont development of *WZSP* tooth germs in mouse subrenal capsules. Pig tooth germ at cap stage could restore diphyodont development and maintain efficient long-term survival and growth in mouse subrenal capsules, which is suitable for future manipulation of large mammalian tooth development.

Tooth agenesis is one of the most common developmental anomalies in humans. Although significant efforts have been made, the mechanism underlying dental agenesis and the molecular regulation of human successional tooth formation remain largely unknown. The mouse as the main mammalian model for tooth development differ significantly from humans, regarding the number, size and shape of teeth, especially in diphyodont and heterodont dentition, which is a less informative model for tooth replacement. Seeking an alternative to mouse model is required for studying diphyodont development and replacement. Compared with mice, pigs (*Sus scrofa*) serve as a promising large animal model for studying human diseases and preclinical therapies owing to its comparability with humans in many respects (Ekser et al., 2012; Groenen et al., 2012).

The generation of successional teeth is derived from a successional dental lamina that forms on the lingual side of the corresponding primary tooth germ. The most common abnormalities of tooth number in human are seen in the permanent dentition. So, abnormalities of successional dental lamina are potentially implicated in the regulation of successional tooth formation. Rare genetic diseases provide valuable insights into human gene function. Some candidate genes are thought to be associated with agenesis and formation of extra teeth (Juuri and Balic, 2017; Phan et al., 2016; Yin and Bian, 2015). Some patients with SOX2 anophthalmia syndrome caused by mutations in the *SOX2* gene are complicated with a dental anomaly and multiple supernumerary teeth (Chacon-Camacho et al., 2015; Numakura et al., 2010). BMP4 is an important member of the TGFβ superfamily and participates in tooth development. The polymorphism of BMP4 was found to be associated with tooth agenesis in human (Gong et al., 2015). It is well known that the activation of canonical Wnt signaling in the dental epithelium is able to induce supernumerary teeth (Lan et al., 2014). The mutations in WNT10B in Chinese families affect the development of permanent dentition and result in oligodontia, by attenuated Wnt signaling in endothelial differentiation of dental pulp stem cells (Yu et al., 2016). Wnt5a activates the β-catenin-independent pathways. Supernumerary teeth were found in patients with dominant Robinow syndrome (DRS), an extremely rare genetic disorder characterized by short-limbed dwarfism, abnormalities in the head, face and external genitalia and dental abnormalities (Mazzeu et al., 2007). Transcription factors participate in epithelial-mesenchymal interactions regulating tooth initiation and morphogenesis (Balic and Thesleff, 2015; Zhang et al., 2005). The dental lamina expresses specific transcription factors which determine the identity and odontogenic potential of the dental mesenchyme (Balic and Thesleff, 2015). Pitx2, the most specific marker of dental epithelium, together with other transcription factors in the dental lamina (such as Foxi3, Dlx2, Lef1 and p63), may be involved in the acquisition of tooth fate and odontogenic potential in the oral epithelium (Balic and Thesleff, 2015). Our results showed specific expression pattern of transcription factors (*Lef1*, *Pitx2*, *Dlx2* and *Msx2*) and signaling molecules (Bmp4 and Wnt10b) in successional dental lamina and p4, implying the possibility of them being related to regulation of successional dental lamina fate and spatiotemporal relationship between primary tooth and their successional counterpart. Further study will contribute to understanding the tooth agenesis or supernumerary teeth.

Previous studies have proven that *WZSP*s can serve as an attractive alternative to rodents for understanding tooth development and replacement (Buchtová et al., 2012; Štembírek et al., 2010; Wang et al., 2014a, 2007). Furthermore, recent significant advances in the pig genome offer a chance to understand the mechanisms underlying tooth development in large mammals (Deng et al., 2011; Ekser et al., 2012; Fan et al., 2013; Fang et al., 2012; Groenen et al., 2012; Hauschild et al., 2011; Li et al., 2014; Matsunari et al., 2013; Walters et al., 2012).

We also tested an alternative to organoids formed from single tooth germ cells of *WZSP in vitro* 3D culture for studying the initiation of early diphyodont development. These alternative methods substantially reduce the overall number of large animals needed, are promising tools to understand diphyodont development for *WZSP* model, and highlight organoid as sources of tissue for potential regeneration of human tooth. Those alternatives can potentially be used to test the mechanism about tooth agenesis and formation of extra teeth.

It is more difficult to study diphyodont development process in pig. Dynamic tracking diphyodont development has been severely hampered by its long growth stage, large size and embryos isolation. There is an increasing demand to seek an alternative environment to maintain growth and long-term tracking of pig tooth germs *in vivo*. In the present study, alternative methods were used to characterize *WZSP*’s diphyodont development. Our results indicated that the microenvironment under renal capsules was sufficient to maintain the survival and growth of intact swine tooth germs required for a longer growing period, and at the same time contributed to generating and tracking the successor tooth germ, which provides the possibility for further research on the molecular and cellular mechanisms governing pig tooth development and replacement using this method.

Our pilot study offers a reference for manipulating tooth germ cells *in vitro* and *in vivo* in *WZSP*s which hold great promise as large animal models for understanding diphyodont development and replacement, and as an organ source for xenotransplantation therapies.

## MATERIALS AND METHODS

### Animals

Ten Pregnant *WZSPs* were obtained from the Institute of Animal Science of the Chinese Agriculture University (Beijing, China). The *WZSP* embryos were obtained as reported previously (Wang et al., 2014a). Briefly, the pregnant *WZSP*s were verified by B-type ultrasonic inspection, and the staged *WZSP* embryos were obtained by cesarean section.

The adult host immunocompromised (SCID) mice (5-week-old) were obtained from the Institute of Laboratory Animal Science, Chinese Academy of Medical Sciences, and maintained in a specific pathogen-free animal facility with free access to water and food.

All experimental animal procedures were reviewed and approved by the Animal Care and Use Committee of Capital Medical University (Permit Number: CMU-2012-x-102), and the methods were carried out in accordance with the approved guidelines.

### Isolation of tooth germs from *WZSP*s

According to our previous research (Wang et al., 2014a,b), the staged *WZSP* embryos and fetuses at E40 were obtained by cesarean section. We chose p4 germs in mandible. The p4 germs in mandibles from the same litter of staged *WZSP* embryos were isolated and pooled under stereo microscopy with an attached Olympus DP72 digital camera system (Olympus Corporation). The morphological stages of the p4 at E40 corresponded to the cap stage and were verified by serial histological sections as previously described (Wang et al., 2014a) (Fig. S1).

### Dissociation and identification of single tooth germ cells

Single tooth germ cells from *WZSP*s were obtained as previously reported (Hu et al., 2005; Ikeda et al., 2009). Briefly, the epithelium and mesenchyme of isolated lower deciduous molar germs were incubated in PBS containing Dispase II (1.2 U ml^−1^, Sigma-Aldrich) and DNase I (20 U ml^−1^, Takara Bio) for 15 min at room temperature, then separated under a stereo microscope. The epithelium and mesenchyme were each dissociated into single cells in PBS (-) supplemented with Collagenase type I (3 mg ml^−1^, Sigma, Worthington Biochemical Corp.) and Dispase II (4 mg ml^−1^, Sigma, Roche Diagnostics Corp.) and filtered through a 70 μm cell strainer (BD Biosciences).

### Tracking the reciprocal interaction between epithelial and mesenchymal cell aggregates and odontogenesis in 3D suspension culture

To track the interaction between epithelial and mesenchymal cells, single cells of mesenchymal origin were transfected with conditional retroviral supernatants from the stable retrovirus-producing cell line PG13/eGFP, as described previously (Wei et al., 2013). Next, single mesenchymal cells marked with eGFP and single epithelial cells were seeded separately in ultra-low-attachment 12-well plates (Corning Costar Corporation) at 1×10^6^ cells ml^−1^ density. They were cultured in medium with DMEM (GIBCO) supplemented with 20% fetal calf serum (GIBCO), 100 U ml^−1^ penicillin/streptomycin, and 0.18 mg ml^−1^ L-ascorbic acid (Sigma-Aldrich) to allow highly efficient formation of spheroid cells. Finally, both cell spheroid types were mixed together in suspension culture to track the interaction of cell aggregates. A 1:1 ratio of mixed cells served as a control (Fluri et al., 2012).

The formed organoid with compartment contact between the epithelial and mesenchymal cell spheroids was injected into a 50 μl collagen gel drop (Cellmatrix type I-A, Nitta gelatin). The collagen gel drop containing organoid was placed on a cell culture insert (0.4-μm pore size, BD, Franklin Lakes) in 12-well cell culture plate, and were cultured for 3 to 14 days in DMEM medium (GIBCO) supplemented with 10% FCS (GIBCO), 100 μg ml^−1^ ascorbic acid (Sigma-Aldrich), 2 mM L-glutamine (Sigma-Aldrich).

### Transplantation of explants into subrenal capsules of SCID mice

The intact deciduous molar germs cultured *in vitro* for 3 days were transplanted into subrenal capsules of adult SCID mice. All mice received the same standardized diet and none of the SCID mice showed any sign of disability after implantation. The host mice were euthanized after 2 or 16 weeks, and perfusion fixed with 4% neutral paraformaldehyde.

### Histochemical and immunohistochemical analysis

For the histology study, the specimens were harvested, fixed in 10% neutral buffered formalin, decalcified, and then embedded in paraffin for preparation of serial sections (5 μm thick). The sections were stained with Hematoxylin-Eosin (H&E) and examined with light microscopy.

For immunohistochemistry of regenerated tooth, briefly, after the samples were fixed with 4% PFA, decalcified, dehydrated and embedded in paraffin, they were cut into 10 μm thick sections. Serial sections were permeabilized in 0.4% Triton X-100 and blocked in PBS containing 5% BSA. Sections were incubated with primary antibodies and overnight at 4°C, then washed and incubated for 1 h at 37°C with the respective secondary antibodies followed by Hematoxylin. The primary antibodies were as follows: anti-SOX2 (ab79351, Abcam, 1:200), anti-BMP4 (ab39973, Abcam, 1:100) and anti-WNT10b (ab66721, Abcam, 1:200). Slices were analyzed using a microscope (BX43 Olympus) with an attached Olympus DP72 digital camera system.

### *In situ* hybridization

The procedure for non-radioactive *in situ* hybridization has been described previously (Wang et al., 2017). Briefly, RT-PCR was performed using mRNA from tooth germ of *WZSP*s. The correct size bands were extracted from agarose gels and DNA sequencing was performed. The primers used for pig *Pitx2*, *Msx2*, *Left*, *Dlx2* were listed in Table S1. The RNA probe was made by labeling with digoxigenin-UTP by *in vitro* transcription with T7 RNA polymerase according to the protocol of DIG RNA labeling Mix (Roche). For the staining procedure, mandible samples were rinsed in RNAse-free PBS and fixed in 4% paraformaldehyde in PBS (pH 7.5). The fixed tissues were decalcified, embedded in paraffin and cut into slices (6 μm). After the rehydration, the slides were treated with proteinase K (1 μg ml^−1^ in PBS) for 30 min at 37°C, and then re-fixed with 4% paraformaldehyde in PBS then rinsed with PBS. The specimens were then dehydrated with series of ethanol, before leaving the slides to air dry for 1 h. The specimens were hybridized in hybridization buffer at 70°C overnight. After washing for 3–4 h, specimens were incubated with alkaline phosphatase conjugated anti-digoxigenin Fab (Roche) overnight. Positive signals were detected by incubating the specimens with NBT/BCIP substrates (Promega).

## Supplementary Material

Supplementary information
