# Supplementary material for: Tracking diphyodont development in miniature pigs in vitro and in vivo
Source: Biol Open. 2019 Jan 25;8(2):bio037036. doi: 10.1242/bio.037036 (PMC6398454; doi:10.1242/bio.037036)
Supplement: Supplementary information [file biolopen-8-037036-s1.pdf]

Tables S1. Primers of genes for in situ hybridization

Gene Name	Sense Primer	Antisense Primer
Lef1,	tcaccctgaagaggaaggtg	cccgtgatgggatatacagg
Pitx2	catgtccacgcgtgaagaaa	ctcgagttacacgtgtccct
Dlx2	tctttgacagtctggtggct	ctaatacgactcactatatcaccgctcttccacatctt
Msx2	cgctcatgtcggacaagaag	ctaatacgactcactatatgggaagcacaggtctatgg

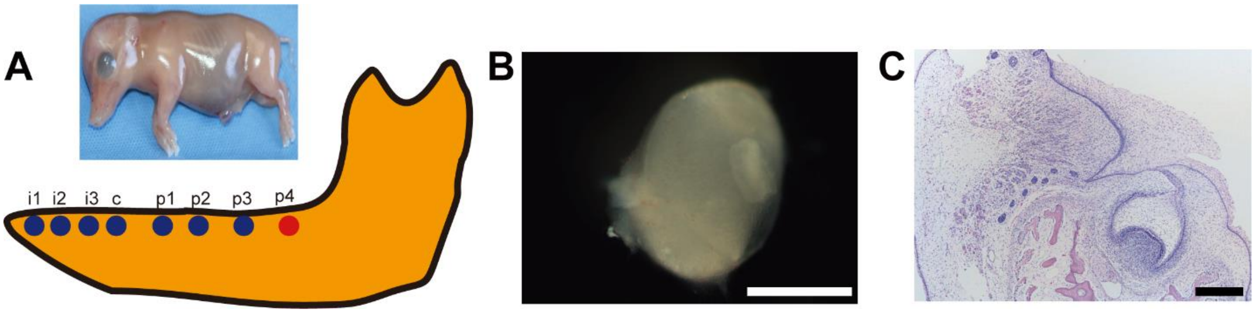

Fig. S1. Isolation of deciduous molar germ from miniature pigs. (A) *WZSP* embryo and schematic buccal view of the mandible of *WZSP* at E40. **(B)** The isolated forth deciduous molar germ (p4) at E40. **(C)** Frontal sections showing p4 at cap stage (H&E). Scale bar = 500 μm.

Biology Open • Supplementary information
